# Paper Strip and Ceramic Potentiometric Platforms Modified with Nano-Sized Polyaniline (PANi) for Static and Hydrodynamic Monitoring of Chromium in Industrial Samples

**DOI:** 10.3390/molecules25030629

**Published:** 2020-01-31

**Authors:** Saad S. M. Hassan, Ayman H. Kamel, Abd El-Galil E. Amr, M. Abdelwahab Fathy, Mohamed A. Al-Omar

**Affiliations:** 1 Chemistry Department, Faculty of Science, Ain Shams University, Abbasia, Cairo 11566, Egypt; Dr_ma7moud@icloud.com; 2 Pharmaceutical Chemistry Department, Drug Exploration & Development Chair (DEDC), College of Pharmacy, King Saud University, Riyadh 11451, Saudi Arabia; malomar1@ksu.edu.sa; 3 Applied Organic Chemistry Department, National Research Center, Dokki, Giza 12622, Egypt

**Keywords:** chromium^III^ assay, potentiometric sensors, paper and ceramic substrates, polyaniline, chronopotentiometry, impedance spectroscopy

## Abstract

Screen-printed membrane sensors based on the use of paper and ceramic substrates are fabricated, characterized, and used for rapid batch and continuous monitoring of Cr^III^ in the form of CrO_4_^2−^ in some industrial products and wastewater samples. Strips of paper and ceramic platforms (15 × 5 mm) were covered with conductive carbon paint and then modified with polyaniline (PANI) film, to act as an ion-to-electron transducer, followed by a drop casting of plasticized poly (vinyl chloride) (PVC) Rhodamine-B chromate membrane as a recognition sensing material. In a 5.0 mmol L^−1^ Trizma buffer solution of pH ~8, the fabricated paper and ceramic based membrane sensors exhibited a near Nernstian response for Cr^VI^ ion with slopes of −29.7 ± 0.5 and −28.6 ± 0.3 mV decade^−1^, limit of detection 2.5 × 10^−5^ and 2.4 × 10^−6^ mol L^−1^ (1.3–0.12 µg mL^−1^), and linear concentration range 7.5 × 10^−3^–5.0 × 10^−5^ and 7.5 × 10^−3^–1.0 × 10^−5^ mol L^−1^ (390-0.5 µg mL^−1^), respectively. Both sensors exhibited fast and stable potentiometric response, excellent reproducibility, and good selectivity with respect to a number of common foreign inorganic species. Impedance spectroscopy and chronopotentiometry data revealed a small resistance and a larger double layer capacitance due to the presence of the intermediate polyaniline (PAN) conductive layer. Furthermore, the formation of a water layer between the ion selective membrane (ISM) and the underlying conductor polymer and between the conducting polymer and the carbon conducting surface was greatly reduced. The developed disposable solid-contact potentiometric sensors offer the advantages of simple design, long term potential stability, flexibility, miniaturization ability, short conditioning time, and cost effectiveness that enable mass production. The sensors were successfully used for static and hydrodynamic measurements of total chromium in some leather tanning wastewater and nickel-chrome alloy samples. The results compare favorably with data obtained by atomic absorption spectrometry.

## 1. Introduction

Chromium metal occurs in the environment in trivalent and hexavalent ionic forms. Chromium (III) ion is essential to humans and other animals due to its role in glucose and cholesterol metabolism, whereas Cr (VI) has many industrial applications and is very toxic [1,2]. Occupational exposure to chromium occurs from chromate processing, stainless-steel production, chrome plating, and tanning industries [3]. The chemical speciation of chromium in environmental samples is necessary for accurate assessments of the pollution source and levels [4,5]. The methods cited in the literature for the determination of both Cr(VI) and Cr(III) include spectrophotometry [6,7], fluorimetry [8,9], inductively coupled plasma/Atomic emission spectrometry (ICP/AES) [10], atomic emission spectrometry [11], atomic absorption spectrometry [12,13], X-ray fluorescence spectrometry [14], differential pulse polarography [15], adsorptive stripping voltammetry [16,17], and high-pressure liquid chromatography [18]. Many of these methods, however, involve several time-consuming manipulation and extraction steps, derivatization reactions, liable to various interferences, not applicable to colored and turbid solutions and require sophisticated instruments.

Potentiometric membrane sensors for the determination of Cr(VI) and Cr(III) have been suggested as an alternative simple technique. These sensors are based on the use of suitable ionophore in plasticized polymeric membranes. Aliquat336 [19], oxalic acid bis (cyclohexylidene hydrazide) [20], 4-dimethylaminoazo-benzene [21], and nickel bathophenanthroline [22] have been suggested as electroactive sensing material for chromium ions in a classical tubular design. The conventional design of these sensors involves direct contact of the inner surface of the sensing membranes with the internal reference solution. This configuration suffers from some problems such as the large size, trivial maintenance, hard portability, and difficulties in both miniaturization and mass production.

The development of coated-wire electrodes (CWEs) as a first example of a solid contact sensor [23] excludes the use of the internal reference solution and enables simple handling and ease of scaling down. However, this type has the main inherent disadvantage of long-term potential stability, probably due to the blocked interface formed between the purely electronic conductor-metal substrate, and the purely ionic conductor polymeric sensing membrane [24]. This problem was solved by using an intermediate layer of suitable material to act as ion-to-electron transducers [25]. Conducting polymers [26,27,28], porous carbon and carbon nanotubes [29] and anion doped nanocluster films [24] have been suggested as suitable material to offer a redox capacitance transduction mechanism. The advantage of using these conducting polymers is the mixed ionic and electronic conductivity and therefore their functions as ion-to-electron transducer with well-defined equilibria at the sensor membrane.

In this work, solid contact Cr(VI) membrane sensor involving the use of cheap and easily prepared polyaniline (PANI) as a conducting polymer film inserted between Rhodamine-B/Cr (VI)/PVC sensing membrane and the conducting base was prepared and examined. Instead of using metal wires commonly utilized in coated wire sensors, carbon printed paper strip and ceramic platform were used. The electrochemical properties of these sensors were examined and characterized by chronopotentiometry and impedance spectroscopy. The proposed sensors exhibit good potential response with high stability and reproducibility and are satisfactorily used for batch (hydrostatic) and continuous (hydrodynamic) measurements of chromium (VI) and chromium (III) in some environmental and industrial samples.

## 2. Results and Discussions

Localized spot area on a paper strip and ceramic platform (15 × 5 mm) were screen printed with carbon ink, followed by depositing thin film of polyaniline as a solid contact and coated with plasticized poly(vinyl chloride) containing Rhodamine-B/ Cr(VI) sensing membrane. These devices were examined and characterized to show the effect of each component and used as potentiometric sensors for Cr(VI). The use of polyaniline as a solid-contact avoided many of the drawbacks commonly observed with coated wire sensors and similar devices including the slow rate of electron exchange and irreversible response toward the nature and concentration of the analyte [30]. The conducting PANI layer induced ion-to-electron transduction (i.e., coupling of ion and electron transfer in the membrane) based on the mixed ionic and redox sensitivity of it. Due to its electronic conductivity, PANI is expected to facilitate both the electron transport in the membrane and the electron transfer at the substrate-membrane interface. This interface can be simply described as an asymmetrical electrical capacitor [30]. The interfacial potential at the ion selective membrane/solid contact interface can be explained by ion partitioning between two phases.

### 2.1. SEM and EDAX Measurements

**Figure 1:**
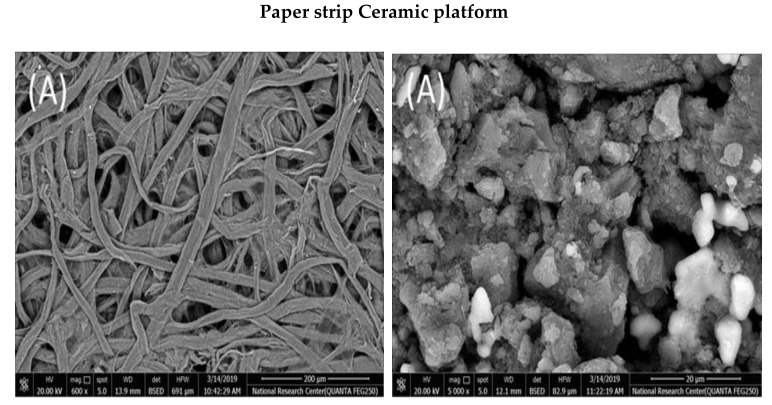

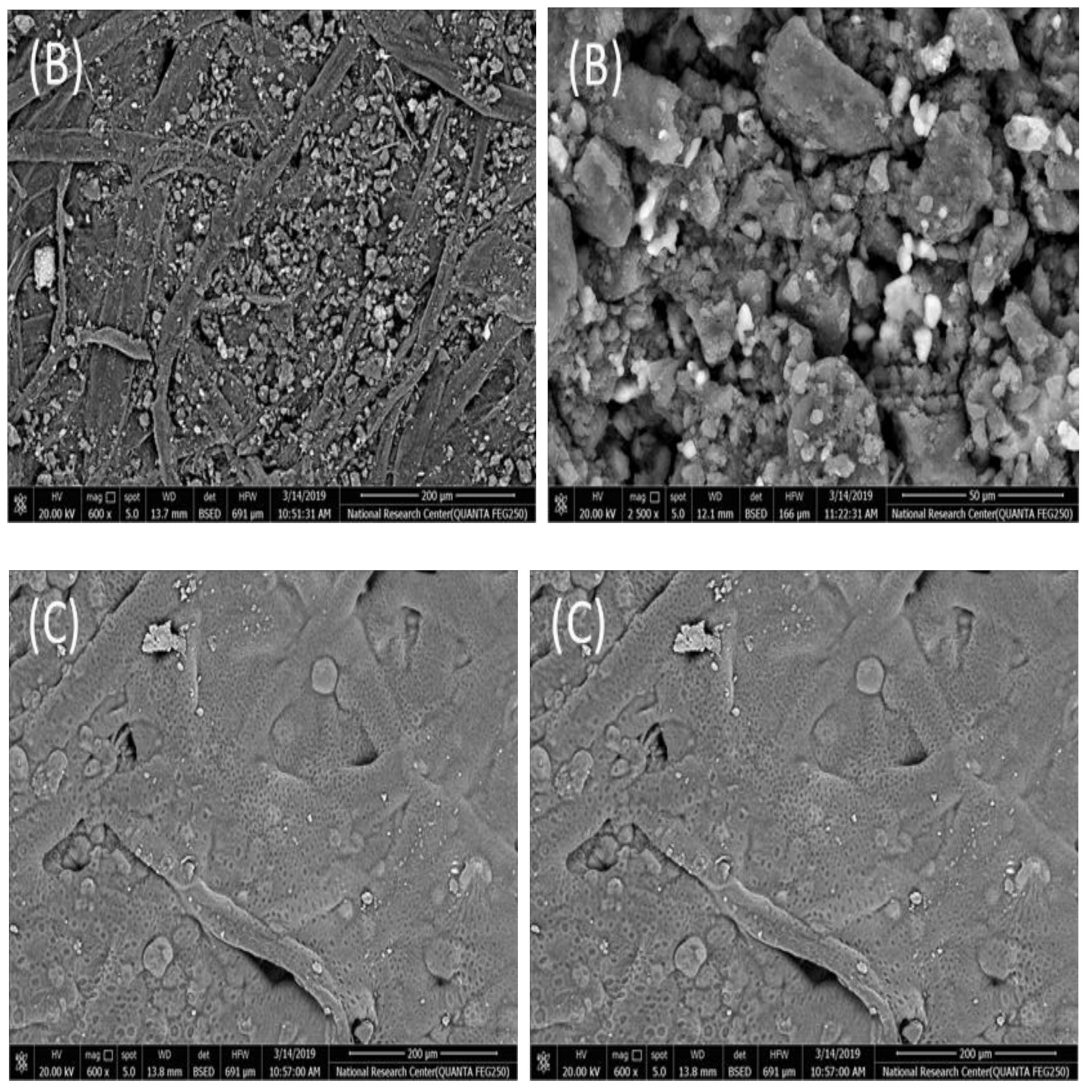
SEM images of the paper and ceramic substrates treated with (**A**) Conductive carbon; (**B**) Conductive carbon + polyaniline (PANI); and (**C**) Conductive carbon + PANI + poly (vinyl chloride) (PVC) sensing membrane.

The morphology of carbon printed paper strip and ceramic platform with and without PANI coating film and PVC chromium (VI) sensing membrane were examined using FESEM and the typical images are presented in Figure 1. Appreciable differences in the morphology of each strip with and without PANI were noticed. The conductive carbon entered and covered pores of the paper strip and ceramic-based platform leading to high conductivity. Deposition of PVC chromium (VI) sensing membrane on PANI coated substrate revealed very good coverage of the exposed sensor area and displayed significant adhesion of the sensing material with the PANI coated paper and ceramic substrates.

### 2.2. Potentiometric Response

**Figure 2:**
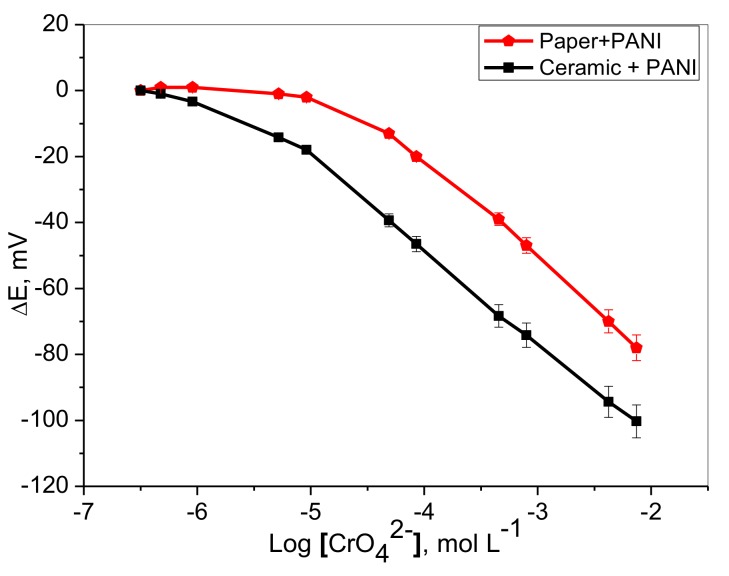
Calibration plots of paper and ceramic based chromium(VI) sensors with PANI solid contact in 5.0 mmol L^−1^ Trizma buffer of pH ~8.

Preliminary experiments using the developed planar chromium (VI) sensors in conjunction with Ag/AgCl reference electrode showed that the sensor potential (mV) decreases linearly with logarithmic Cr(VI) concentration in 5.0 mmol L^−1^ Trizma buffer of pH ~8. The typical dynamic potentiometric response characteristics and calibration plots are shown in Figure 2. Although both types of sensors exhibited similar rapid linear response for chromium(VI), a paper strip-based sensor with a PANI layer showed linear response in the range of 7.5 × 10^−3^–5.0 × 10^−5^ mol L^−1^ (390–2.6 µg mL^−1^) with near Nernstian slopes of −29.7 ± 0.5 (*r^2^* = 0.997) mV decade^−1^, and lower detection limit of 2.5 × 10^−5^ mol L^−1^ (1.3 µg mL^−1^), respectively.

On the other hand, the potential of the ceramic platform based sensor with PANI layer displayed a linear response for chromium (VI) over the concentration range of 7.5 × 10^−3^–1.0 × 10^−5^ mol L^−1^ (390–0.52µg mL^−1^) with a near Nernstian slope of −28.6 ± 0.3 (r^2^ = 0.9996) mV decade^−1^, and a detection limit of 2.4 × 10^−6^ mol L^−1^ (0.12 µg mL^−1^). These data revealed that the calibration ranges and slopes of both sensors are very close, but the lower detection limit offered by the ceramic based sensor was at least one order of magnitude more sensitive (Table 1).

### 2.3. Effect of pH

**Figure 3:**
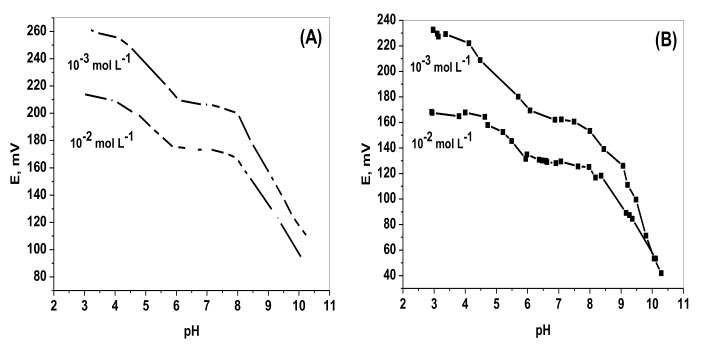
Influence of pH on potentiometric response of: (**A**) Paper, and (**B**) ceramic based Chromium(VI) sensors with PANI solid contact.

In aqueous solutions, chromium (VI) ion exists in different anionic forms (dichromate, Cr_2_O_7_^2−^), (chromate, CrO_4_^2−^), (hydrogen dichromate, HCr_2_O_7_^−^), (hydrogen chromate, HCrO_4_^−^), (trichromate, Cr_3_O_10_^2−^), and (tetrachromate, Cr_4_O_13_^2−^) depending on both the pH and the concentration of chromium. All poly-oxyanions of chromium (VI) are only formed in strong acidic solutions [31,32]. The effect of pH on the sensor response was tested by measuring the sensor response potential for 1.0 × 10^−2^ and 1.0 × 10^−3^ mol L^−1^ chromium (VI) solutions over the pH range 2–10. A constant potential was observed over the pH range 6–8 for both paper and ceramic based chromium(VI) sensors (Figure 3) indicating that CrO_4_^2−^ is the species sensed by the sensors. It has been reported that chromate ion (CrO_4_^2−^) is predominant at pH > 6 (Equation (1)) and become ≈ 100% at a pH = 8 [32].

HCrO_4_^−^ ⇌ CrO_4_^2−^ + H^+^ p*K*_a_ ≈ 5.9 (1)

Chromate and dichromate ions are also present in equilibrium, although chromium ion is present in both as Cr (VI). This equilibrium (Equation (2)) does not involve a change in hydrogen ion concentration, which would predict that the equilibrium is independent of pH [31,32].

2 HCrO_4_^−^ ⇌ Cr_2_O_7_^2−^+ H_2_O (2)

### 2.4. Chronopotentiometric Measurements

**Figure 4:**
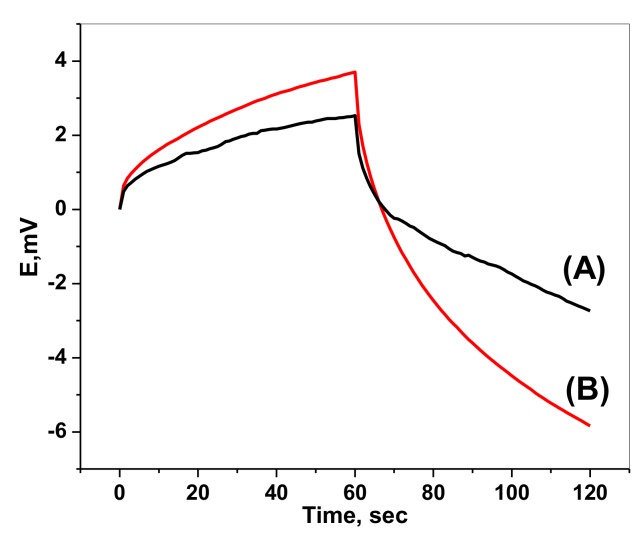
Chronopotentiograms of paper based sensor for chromium (VI) sensors (**A**) with PANI as a solid contact and (**B**) without using PANI, applied current: +1 nA for 60 s and −1 nA for 60 s.

To evaluate the short-term potential stability of the developed paper based sensors, chronopotentiograms were recorded under a constant current of ±1 nA and the results are shown in (Figure 4) [33]. As can be seen, in the absence of PANI, a large potential polarization up to 89.2 ± 2.5 µV s^−1^ was obtained. In the presence of PANI, a smaller potential drift value of 44 ± 1.6 µVs^−1^ was recorded. The improved potential stability of PANI based sensor is probably due to the redox capacitance of PANI as confirmed by applying the fundamental equation (Equation (3)).

∆E/∆t = i/C(3)

where ∆E, ∆t, i, and C represent the change of potential, variation of time, applied current and capacitance, respectively. The capacitance was found to be 22.7 ± 1.3 and 11.2 ± 1.6 µF in presence and absence of PANI layer. This demonstrates good potential stability of the developed solid contact based sensor.

### 2.5. Water Layer Test

The effect of water film formation on the potential drift due to membrane adhesion on, and membrane delamination from the underlying conductor in an alkaline media usually militates against obtaining a stable potential of the sensors. A water layer present between the PVC recognition sensing membrane and the underlying conductor has already been reported, and the existing water layer causes problems of mechanical failure, responsive hysteresis, and potential instability [34]. To confirm the presence or absence of a water layer in the proposed sensors, the simple test protocol previously proposed [34] was utilized. In the presence of a water layer, potential drifts would be observed by replacing the primary ion solution with an interfering ions solution and vice versa. Typically, a positive potential drift was observed upon changing of the measured solution from primary ions to interfering ions, while a negative potential drift was obtained upon replacing the interfering ions with primary ions. These asymmetric characteristics are due to the change of the composition of water layer as a result of transporting of some ions from the samples and consequently diffuse through the ion sensing membrane.

**Figure 5:**
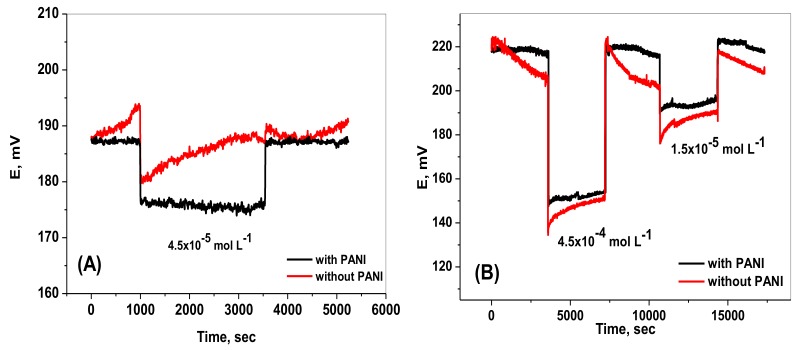
Potential drift due to formation of water layer between (**A**) paper and (**B**) ceramic based chromium(VI) sensors with a PANI solid contact layer and the sensing PVC membrane.

For a sensor based on both paper and ceramic substrates, the water layer test was carried out by sequential immersions of paper strips with and without PANI in a 5.0 mmol L^−1^ Trizma buffer (pH ~8) solution for the first hour, in a 4.5 × 10^−4^ mol L^−1^ chromate solution for the second hour, in 5.0 mmol L^−1^ Trizma buffer (pH ~8) solution for the third hour, then in 1.5 × 10^−5^ mol L^−1^ Cr(VI) solution, and finally in 5.0 mmol L^−1^ Trizma buffer (pH ~8) solution. For the paper-based sensor, the water layer test was carried out by sequential immersions of paper strips with and without PANI in 5.0 mmol L^−1^ Trizma buffer (pH ~8) solution for 15 min, in 4.5 × 10^−4^ mol L^−1^ chromium(VI) solution for 30 min, then in 5.0 mmol L^−1^ Trizma buffer (pH ~8) solution for 30 min. The results are illustrated in Figure 5. In the absence of PANI, a significant potential shift and negative potential drift were noticed. Upon changing the chromium (VI) solution to 4.5 × 10^−4^ mol L^−1^, a positive potential drift occurred during the second hour. The same potential drift occurred upon using 1.5 × 10^−5^ mol L^−1^ of chromium (VI) solution. However, in the presence of the PANI layer, nearly no significant potential drifts were observed upon changing the chromium (VI) solution from 4.5 × 10^−4^ to 1.5 × 10^−5^ mol L^−1^. These results confirm that minimal water layer was formed between the PANI solid-contact layer and the sensor recognition membrane, probably due to the high hydrophobicity of the PANI layer.

### 2.6. Sensor Selectivity

**Figure 6:**
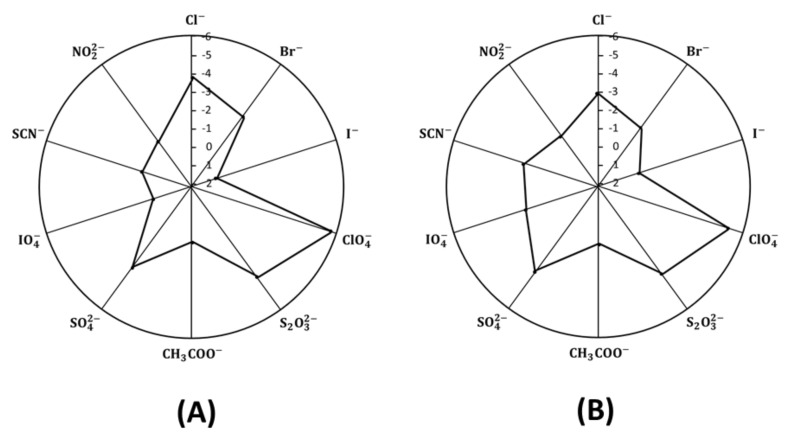
Selectivity coefficient diagrams of: (**A**) paper and (**B**) ceramic based chromium(VI) sensors with PANI solid contact using the modified separate solutions method (MSSM).

Selectivity coefficients log*K^pot^_CrO42-,J_* of the proposed paper and ceramic based chromium(VI) sensors were determined by using the modified separate solutions method. A fresh sensor with membrane that has never been in contact with the primary chromium (VI) ions was used. The measurement of selectivity was started with the most discriminated (less interfering) ions. This allows the relatively preferred ions to readily displace the initially contained ions in the membrane and, thus, to completely saturate the membrane [35]. The highest measured concentration (10^−1^ mol L^−1^ was employed for the determination of selectivity with the established formalisms [36]. The single ion activities were calculated by the extended Deby–Huckel equation. The potentiometric selectivity coefficient values are shown in Figure 6. The selectivity sequence of paper based membrane sensors with solid PANI contact was found to be in the order: CrO_4_^2−^> SCN− > CH_3_COO^−^ > NO_2_^−^ > IO_4_^−^ > I− > Br^−^> SO_4_^2−^> Cl^−^ > S_2_O_3_^2−^ > ClO_4_^−^. For ceramic SPE membrane based sensor, the selectivity order was in the order: CrO_4_^2−^ > SCN^−^ > NO_2_^−^ > CH_3_COO^−^ > I^−^ > Br^−^ > Cl^−^ > S_2_O_3_^2−^ > SO_4_^2−^ > ClO_4_^−^. This selectivity order clearly differs from the classical Hofmeister pattern. This can be attributed to the properties of the ion association complex used as an ionophore. Generally, the selectivity coefficient values of the paper based chromium (VI) sensor were much better than those of the ceramic based chromium (VI) sensor.

### 2.7. Batch and Continuous Determination of Chromium (III) and Cr(VI)

Paper strip and ceramic platform solid contact chromium (VI) membrane sensors were used for determining Cr(VI) under batch mode of operation. These sensors were also utilized as detectors for continuous flow-injection analysis (FIA) of Cr(VI) and Cr(III) ions, after oxidation. Under batch measurement, 10 µg mL^−1^ control chromium (VI) sample showed results with an average recovery of 99.8 ± 0.5% and a relative standard deviation of ±1.4%. The potential readings were reproducible within ±0.7 mV and stable for at least 30 days. For hydrodynamic mode of operation (FIA), a preliminary study was made to investigate some parameters affecting width, height, and resolution of the peaks and sample output. Different carrier flow rate (2–7 mL min^−1^) and sample loop volumes (100–500 µL) were tested.

**Figure 7:**
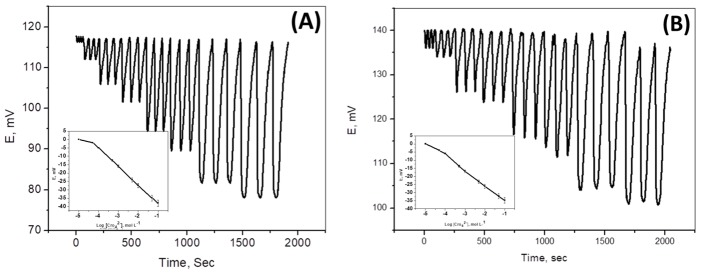
Typical transient flow-injection analysis (FIA) potentiometric signals using: (**A**) Paper based and (**B**) ceramic based chromium(VI) sensors with PANI solid contact; (Conditions: carrier solution, 5.0 × 10^−3^ mol L^−1^ Trizma buffer of pH ~8, flow rate 4.0 mL min^−1^; sample volume, 100 µL).

Optimum peak width, washing time, recovery time, sample frequency, residence time, and absence of memory effect were obtained by using a 5.0 × 10^−3^ mol L^−1^ Trizma buffer of pH ~8 as a background at a flow rate of 4 mL min^−1^ and loop volume of 100 µL. Under optimized conditions, a linear response (r^2^ = 0.9998) was obtained between FIA readout in mV and logarithm of chromium (VI) concentration (mol L^−1^) over the range 1.0 × 10^−1^–4.9 × 10^−5^ mol L^−1^ (5100–2.5 ppm). The detection limit was 3.5 × 10^−5^ mol L^−1^ (1.8 ppm). A control sample containing 10 µg mL^−1^ Cr (VI) showed an average recovery of 98.1 ± 1.1% and a mean standard deviation of ±0.7%. The potential response was stable and reproducible within ±1.6 mV for at least 30 days. The sampling frequency was 45–50 samples per hour (Figure 7).

### 2.8. Determination of Chromium in Some Environmental and Industrial Samples

The chromium contents of some leather tanning wastewater and Ni-chrome heating wires were potentiometrically determined using the proposed paper and ceramic based sensors under batch and hydrodynamic (FIA) modes of measurements. The samples were acidified with dilute HCl, followed by oxidation with alkaline hydrogen peroxide (30%) to convert Cr(III) into Cr(VI) (Equation (4)) [37] before assessment of the total chromium [Cr(III) + Cr(VI)] by the proposed sensors. Parallel runs were performed using flame atomic absorption spectrometry for comparison.

(4) $$2\mathrm{Cr}(\mathrm{OH}{)}_{3}\;+\;4\;{\mathrm{OH}}^{-}\;+\;3H_{2}O_{2}\;\to 2{\left(\mathrm{Cr},O_{4}\right)}^{2-}+8H_{2}O$$

The average recoveries of total chromium in some leather tanning wastewater samples collected from local factories and measured by potentiometric batch and continuous mode of operations with the paper based sensors were 99.5 ± 0.7% and 100.0 ± 0.7% (*n* = 5), respectively. With ceramic based sensors, average recoveries of 100.0 ± 0.8% and 98.9 ± 0.8%, were obtained by using batch and continuous operation technique, respectively (Table 2).

Chromium contents in some nickel-chrome heating wires and alloys were similarly measured by sample dissolution in concentrated hydrochloric acid, dilution, treatment with hydrogen peroxide and potentiometric assessment using the standard addition method to overcome the interference caused by the presence of residual chloride ions. The results obtained with paper and ceramic chromium (VI)-based sensors using batch experiments (*n* = 5) showed average recoveries of 95.9 ± 1.2% and 101.8 ± 0.9%, respectively. Under hydrodynamic mode of operation, the mean average recoveries were 95.4 ± 1.1% and 106.0 ± 0.9% with the paper- and ceramic-based chromium sensors, respectively (Table 3).

## 3. Materials and Methods

### 3.1. Apparatus

Potential measurements were made at 25 ± 1 °C with an Orion (Cambridge, MA, USA) model 720/SA pH /mV meter using chromium(VI) membrane sensor in conjunction with an Orion Ag/AgCl double-junction reference electrode (model 90–20) filled with 1 mol L^−1^ CH_3_COOLi. A combination Ross glass electrode (Orion 81-02, MA, USA) was used for pH measurements. The flow injection manifold utilized for the analysis of Cr(VI) consisted of a two-channel peristaltic pump (Ismatech Ms–REGLO, MA, USA), polyethylene tubing (0.71 mm i.d.) and an Omnifit injection valve (Omnifit, Cambridge, UK) with a 100 µL sample loop volume. The potential signals were recorded using a home-made high input-impedance 8-channel box connected to a PC through the interface ADC 16 (Pico Tech, London, UK) and Pico Log software (version 5.07, London, UK). Atomic absorption spectrometric measurements were made with a Perkin-Elmer 3100 (Waltham, MA, USA) at 357.9 nm using the standard recommended method [38]. The chronopotentiometry measurements were carried out using a Metrohom potentiostat/galvanostat (Autolab, model 204, Herisau, Switzerland) purchased from Metrohom Instruments (Switzerland) connected to a conventional, three-electrode cell. An Ag/AgCl/3 M KCl/1 M LiOAc was the reference electrode, a Pt rod was used as the auxiliary electrode and the SC-ISE was connected as a working electrode.

The SEM images of the paper and ceramic substrates were examined using the scanning electron microscope (SEM) (JOEL-JSM-IT500HR, Osaka, Japan).

### 3.2. Materials and Reagents

All chemicals and reagents used in this work were of analytical reagent grade unless otherwise stated and doubly distilled water was used throughout. Whatman qualitative filter paper (Grade 5, COTRAN833 (KC63) was used. High molecular weight poly (vinyl chloride) (PVC) and *o*-nitrophenyloctyl ether (*o*-NPOE) were used as received from Fluka (Ronkonoma, NY, USA). Conductive carbon paint was purchased from (Merck, Darmstadt, Germany). Hydrogen peroxide (30%). Tetrahydrofuran (THF), Rhodamine-B (RB), potassium chromate, and Trizma^®^ buffer were purchased from Sigma Chem. Co (St. Louis, MO, USA).

A 1.0 × 10^−1^ mol L^−1^ stock solution of CrO_4_^2−^ was freshly prepared in de-ionized water. Working chromium (VI) solutions (1.0 × 10^−2^–1.0 × 10^−6^ mol L^−1^) were prepared by accurate dilutions, and stored in brown bottles. Rhodamin-B /chromate ion pair complex was prepared by mixing equal volumes of 1.0 × 10^−2^ mol L^−1^ of Rhodamine-B and potassium chromate. The red precipitated complex was collected by filtration, washed with distilled water, dried in air, and ground to fine powders.

### 3.3. Preparation of Solid Contact Cr(VI) Sensor

#### 3.3.1. Fabrication of Paper and Ceramic-Based Sensors

**Figure 8:**
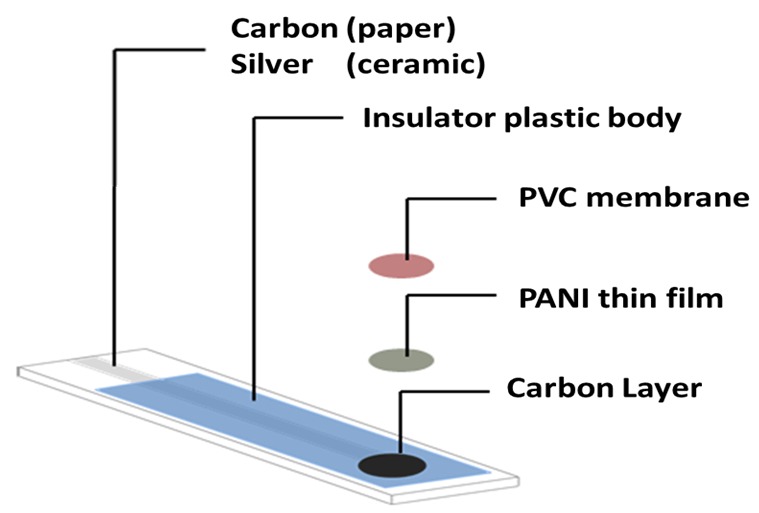
Fabrication of paper and ceramic based chromium (VI) sensors with PANI solid contact.

Paper-based sensor was made by cutting a filter paper into rectangular pieces (15 × 5 mm) and coated on both sides with a conductive carbon paint using a small soft brush. The paper was covered and sandwiched between two insulator plastic sheets (10 × 8 mm). No such plastic sheets were used with the ceramic substrate. One of the plastic sheets used with paper based substrate contains an orifice (4 mm diameter) on its lower side as shown in (Figure 8). The uncovered conductive part of the paper strip was connected through a crocodile to a PNC connector (75 Ohm) which is directly coupled with the reading instrument.

#### 3.3.2. Preparation of Polyaniline (PANI)

Aniline hydrochloride (2.59 g, 20 mmol) was dissolved in the least quantity of distilled water, transferred to a 50 mL volumetric flask and completed to the mark with de-ionized water. Similarly, ammonium peroxydisulfate (5.71 g, 25 mmol) was dissolved in 50 mL water. Both solutions were kept for 1 h at room temperature (18–24 °C), mixed in a beaker, stirred, and left to stand for polymerization and formation of polyaniline (PANI) precipitate. After 24 h, the precipitate was collected on a filter paper, washed with 100 mL portions of 0.2 mol L^−1^ HCl followed by acetone and dried in air [39]. A 3-milligram portion of the prepared PANI was dissolved in 100 μL chloroform and the solution was applied by drop-casting on the orifice existing on the carbon screen printed paper strip or the ceramic platform using a micropipette. The solution was left to dry for 10 min to form a thin film of PANI.

#### 3.3.3. Preparation of Rhodamine-B/ Cr(VI) Membrane Sensor

A 2-mg portion of Rhodamine-B/chromate ion pair complex was thoroughly mixed with 133 mg of *o*-NPOE, 66 mg of PVC and 2 mL of THF solvent. A 20-μL aliquot of this membrane cocktail was drop-casting on the graphite/PANI spot on the paper strip and ceramic platform screen using a micropipette. The solution was left for 2 h for THF evaporation and membrane drying. The sensors were conditioned by soaking in a 1.0 × 10^−2^ mol L^−1^ aqueous CrO_4_^2−^ solution for 12 h.

### 3.4. Electrochemical Measurements

Carbon screen printed filter paper strip and ceramic platform based chromium (VI) sensors in conjunction with a double junction Ag/AgCl reference electrode were immersed in a 25 mL beaker containing 10.0 mL of 5.0 × 10^−3^ mol L^−1^ Trizma buffer of pH ~ 8. Aliquots (0.5–1.0 mL) of standard 1.0 × 10^−1^–1.0 × 10^−5^ mol L^−1^ chromium (VI) solutions were added and the potential readings were recorded after stabilization to ±1 mV. The potential response was plotted as a function of the logarithm Cr(VI). The calibration plot was used for subsequent measurements of unknown chromium (VI) solutions.

### 3.5. Sensor Selectivity

The selectivity coefficients of the proposed sensors were determined by using the modified separate solutions method (MSSM) [40]. The EMF response values of different interfering metal ion solutions in the concentration range of 10^−5^–10^−1^ mol L^−1^ were measured. The selectivity coefficients were calculated according to Equation (5):

Log *K*^pot^_i,j_ = (*E*_*j*_-*E*_*i*_) *z*_*i*_*F*/2.303 *RT* + *loga*_*i*_/*a*_*j*_^*zi/zj*^ (5)

where *E_i_* and *E_j_* are the measured membrane potentials for a solution containing the primary ion *I^z^_i_^+^* and interfering ion *J^z^_J_^+^*, with charges *z_i_* and *z_J_*, respectively. The symbols *F*, *R*, and *T* are Faradays constant, universal gas constant, and temperature, respectively. The activity coefficients were calculated as previously reported [41].

### 3.6. Flow Injection Setup and Continuous Measurements

The used flow injection analysis (FIA) manifold consisted of a two-channel Ismatech MS-REGLO model peristaltic pump and connected with polyethylene tubing (Tygon, 0.7 mm i.d.) with an Omnifit injection valve (Rheodyne, Model 7125). A sample loop of 100 μL volume was used for injection. A carrier solution consisted of 5.0 mmol L^−1^ Trizma buffer (pH ~8) was propelled by means of a peristaltic pump through PTFE tubing (1.13 mm i.d.). The length of the tubing from the injection valve to the detector was 30 cm.

**Figure 9:**
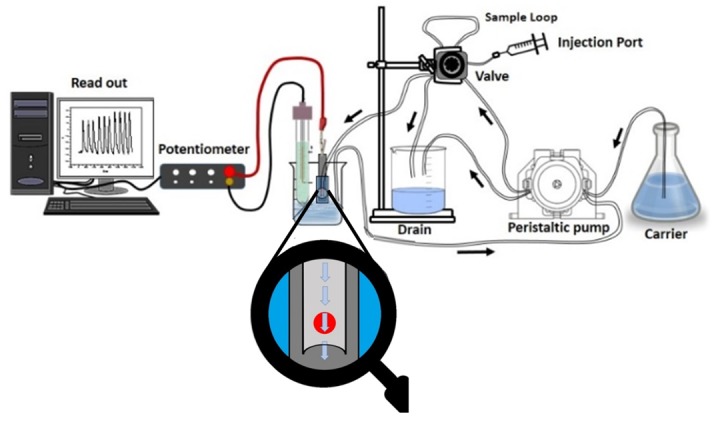
Flow injection setup used for determining Cr(VI) using paper and ceramic based sensors with PANI solid contact.

Paper and ceramic based detectors were constructed by welding the strip-based sensors with a 1.1 mm half circular PVC tube and connected to the exits of PTFE tubing from the injection valve. The half circular PVC tube covered the chromium(VI) sensing membrane as schematically shown in (Figure 9). The sample loop (100 μL) of the injection valve was filled and the valve was rotated to allow the flow of the sample to the detector with the carrier solution (5.0 mmol L^−1^ Trizma buffer, pH ~8). A flow rate of 4.0 mL min^−1^ was used and the signals were recorded using an Orion pH/mV meter (model SA 720, MA, USA) connected to a PC through the interface ADC 16 (Pico Technology, London, UK) and PicoLog for windows (version 5.07, London, UK) software. The average peak height of 3 consecutive replicate runs of each sample was measured and compared with a calibration plot prepared under the same conditions.

### 3.7. Analysis of Real Chromium Containing Samples

#### 3.7.1. Determination of Total Cr (VI) and Cr (III) in Leather Tanning Wastewater

Leather tanning wastewater samples were collected in glass containers from some local leather tanning factories, acidified, and filtered. A 10 mL aliquot of the filtrate was treated with 10 mL of 30% hydrogen peroxide. The pH of the solution was adjusted to ~8 using dilute sodium hydroxide and boiled for 5 min to convert Cr(III) into Cr(VI). After cooling, the solution was transferred to 50 mL volumetric flask and completed to the mark with 5.0 mmol L^−1^ Trizma buffer of pH ~8. The potential of the solution was measured using the developed sensors as described above and compared with the calibration plot. For continuous analysis, a carrier solution consisting of the same buffer and 100 µL aliquots of the test sample were used. Simultaneous determination of Cr(III) and Cr(VI) was performed by measuring the chromium contents of the sample before and after treatment with hydrogen peroxide to indicate [Cr(VI)] and [Cr(III) + Cr(VI)], respectively. The difference corresponded to Cr(III) content.

#### 3.7.2. Determination of Total Chromium Metal in Alloys

A piece of nickel-chrome heating coil wire or chrome alloy (0.59 g) was dissolved in the least volume of concentrated HCl solution. The solution was boiled for complete metal dissolution and to almost dryness. The residue was dissolved in de-ionized water, filtered off, transferred to a 100 mL measuring flask and completed to the mark with 5.0 mmol L^−1^ Trizma buffer of pH ~8. A 20 mL aliquot of the solution was mixed with 20 mL of 30% hydrogen peroxide and the pH was adjusted with dilute sodium hydroxide solution to pH ~8. The mixture was boiled for 5 min, cooled, filtered, transferred to 50 mL volumetric flask and completed to the mark with the buffer. The potential of the solution was measured using the developed sensors and compared with calibration graphs prepared under identical conditions. A blank experiment and atomic absorption spectrometric measurements were carried out in parallel.

The standard addition (spiking) technique was also used [42]. Four aliquots, 2.0 mL each, of the unknown chromium containing solution were transferred to four 10 mL volumetric flask, followed by addition of 0, 1.0, 2.0, and 3.0 mL of standard 1.0 × 10^–2^ mol L^−1^ Cr(VI) solution. The solutions were completed to the mark with Trizma buffer (pH ~8), shaken well and used for batch and continuous chromium measurement using both sensors as described above.

## 4. Conclusions

Novel chromium (VI) planar membrane sensors were developed characterized and used for chromium (VI) and chromium (III) measurements. Paper strip and ceramic platform were utilized as sensor substrate and polyaniline film as a solid contact ion-to-electron transducer. Rhodamine-B/chromium (VI) ionophore dispersed in plasticized poly (vinyl chloride) coated layer was used as a recognition site for chromium (VI). This configuration responded linearly to chromium (VI) ion over the concentration range of 7.5 × 10^−3^–5.0 × 10^−5^ and 7.5 × 10^−3^–1.0 × 10^−5^ mol L^−1^ with Nernstian slopes of −29.7 ± 0.5 and −28.6 ± 0.3 mV decade^−1^ and lower detection limits of 2.5 × 10^−5^ and 2.4 × 10^−6^ mol L^−1^ for paper and ceramic based sensors, respectively. The electrochemical properties of the fabricated sensors in the presence and absence of PANI were systematically investigated. The enhancement effect of PANI on the membrane resistivity, potential stability, and reproducibility were verified by chronopotentiometry and electrochemical impedance spectroscopy. The use of PANI-based solid contact film reduced the water layer between the PANI layer and the sensing membrane. A study of pH effect and the presence of many interfering metal ions showed good performance characteristics of the proposed designs. The sensors were satisfactorily used for rapid static and hydrodynamic (FIA) measurements of chromium (VI) and chromium (III) in some environmental and industrial samples. Results agreed fairly well with atomic absorption spectrometric data were obtained. Significant advantages were offered by the proposed sensors over many of the previously suggested methods.

## Figures and Tables

**Table 1:** Potentiometric response characteristics of paper and ceramic based chromium(VI) sensors with PANI solid contact in 5.0 mmol L^−1^ Trizma buffer (pH ~8).

Parameter	Paper Based Cr(VI) Sensor	Ceramic Based Cr(VI) Sensor
Slope, (mVdecade^−1^)	−29.7 ± 0.5	−28.6 ± 0.3
Correlation coefficient, (r^2^)	0.9977	0.9996
Detection limit, (mol L^−1^)	2.5 × 10^−5^	2.4 × 10^−6^
Linear range, (mol L^−1^)	7.5 × 10^−3^–5.0 × 10^−5^	7.5 × 10^−3^–1.0 × 10^−5^
Working pH range (pH)	6.0–8.0	6.0–8.0
Response time (s)	<10	<10
Repeatability (% mV)	0.9	1.2
Reproducibility (% mV)	1.2	0.8
Accuracy (%)	98.4	99.3

**Table 2:** Potentiometric determination of chromium in some leather tanning wastewater samples using paper and ceramic based chromium (VI) sensors with PANI solid contact.

Sample No.	[Chromium], mg L^−1^				
	AAS	Paper Based Sensor		Ceramic Based Sensor	
		Batch	Flow Injection	Batch	Flow Injection
(1)	71.2 ± 0.4	70.9 ± 0.4	71.1 ± 0.5	72.5 ± 1.0	69.5 ± 0.9
(2)	86.6 ± 0.5	85.9 ± 0.6	86.1 ± 0.6	86.4 ± 0.7	87.1 ± 0.8
(3)	192.2 ± 0.7	191.8 ± 1.1	193.1 ± 0.9	192.8 ± 0.8	189.3 ± 0.7

* Average of 5 measurements.

**Table 3:** Potentiometric determination of chromium in some nickel-chrome heating wires and alloys using paper and ceramic-based chromium (VI) sensors with PANI solid contact.

Sample No.	[Chromium], mg g^−1^				
	AAS	Paper Based Sensor		Ceramic Based Sensor	
		Batch	Flow Injection	Batch	Flow Injection
(1)	197.5 ± 1.2	192.6 ± 1.1	202.5 ± 0.8	195.6 ± 0.7	207.4 ± 0.6
(2)	162.9 ± 1.5	153.1 ± 1.2	148.2 ± 1.5	167.9 ± 1.1	172.8 ± 0.9
(3)	138.3 ± 0.9	133.3 ± 1.0	128.4 ± 1.6	143.2 ± 0.9	148.1 ± 1.1

* Average of 5 measurements.
